# Supplementary material for: Optimizing Ex Vivo CAR-T Cell-Mediated Cytotoxicity Assay through Multimodality Imaging
Source: Cancers (Basel). 2024 Jul 9;16(14):2497. doi: 10.3390/cancers16142497 (PMC11274748; doi:10.3390/cancers16142497)
Supplement: Supplementary file 1 [file cancers-16-02497-s001.zip › Sup_Figures_CAR-T_Assay_20240702_V6.pdf]

Supplementary Materials

Optimizing ex vivo CAR-T cell-mediated cytotoxicity assay through multimodality imaging

John G. Foulke, Luping Chen, Hyeyoun Chang, Catherine E. McManus, Fang Tian* and Zhizhan Gu*

American Type Culture Collection (ATCC), Manassas, VA 20110, USA

*Correspondence: ftian@atcc.org (F.T.); zgu@atcc.org (Z.G.)

Supplementary Video Legends:

Videos 1 and 2: CD19 CAR-T in vitro killing assay of Raji-Luc2 measured using live cell imaging. Raji-Luc2 cells were stained with Vybrant DiO dye and real-time fluorescent images were captured every 30 minutes for 24 hours during co-culture with either CD19 or mock CAR-T cells.

Videos 3 and 4: CD20 CAR-T in vitro killing assay of Farage-Luc2 measured using live cell imaging. Farage-Luc2 cells co-culture with CD20 or mock CAR-T cells in the presence of Incucyte Cytotox red dye. Real-time fluorescent images were captured every 1 hour for 24 hours during the co-culture. Scale bar, 1000 µm.

Videos 5 and 6: CD20 CAR-T in vitro killing assay of Daudi-Luc2 measured using live cell imaging. Daudi-Luc2 cells co-culture with CD20 or mock CAR-T cells in the presence of Incucyte Cytotox red dye. Real-time fluorescent images were captured every 1 hour for 24 hours during the co-culture. Scale bar, 1000 µm.

Supplementary Figures (next pages):

Supplemental Fig. S1

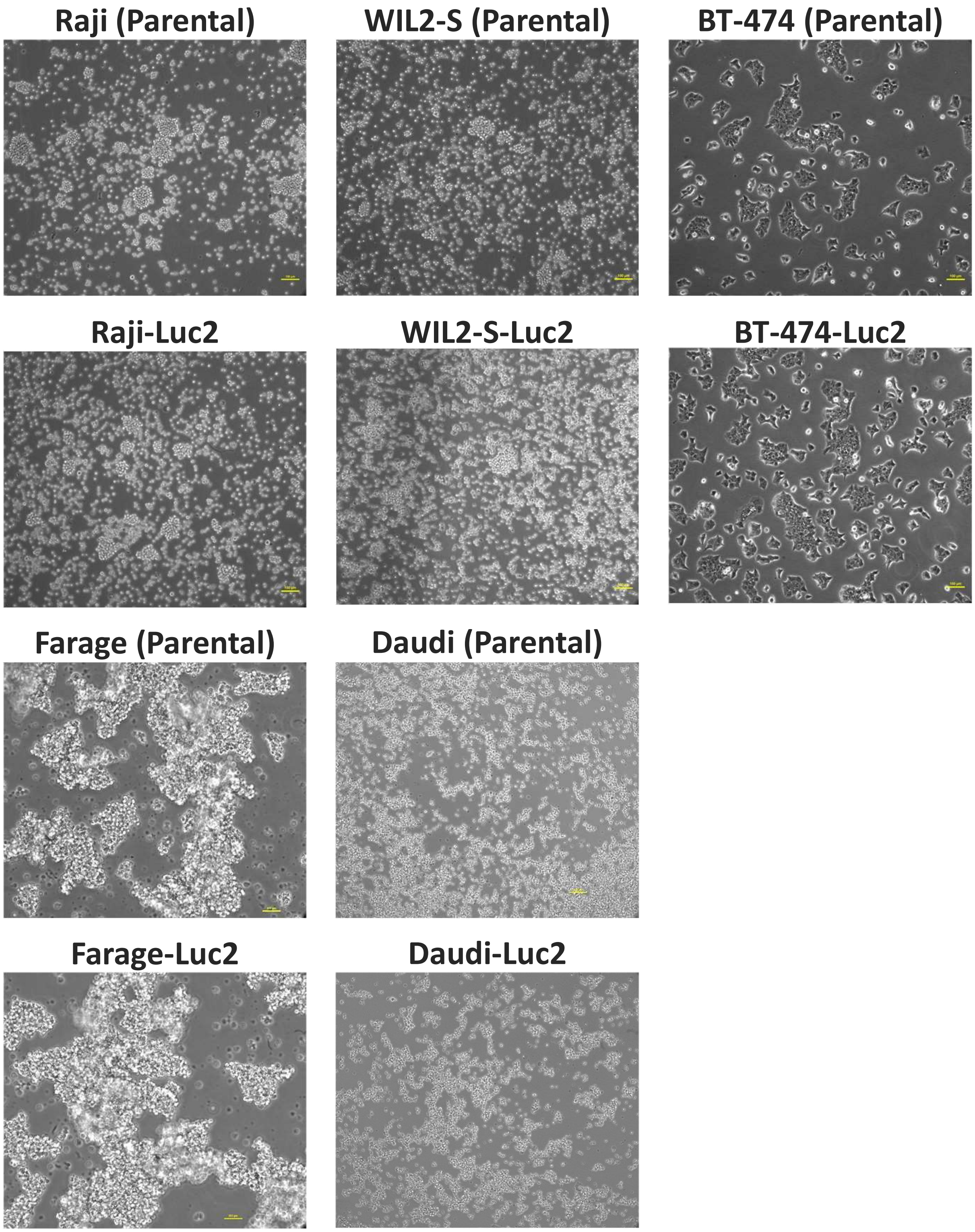

Figure S1: Characterization of CAR-T target cell lines: Cell Morphology. Cell morphology of the luciferase-expressing cell lines compared to their parental lines. Scale bar, 100 µm.

Supplemental Fig. S2

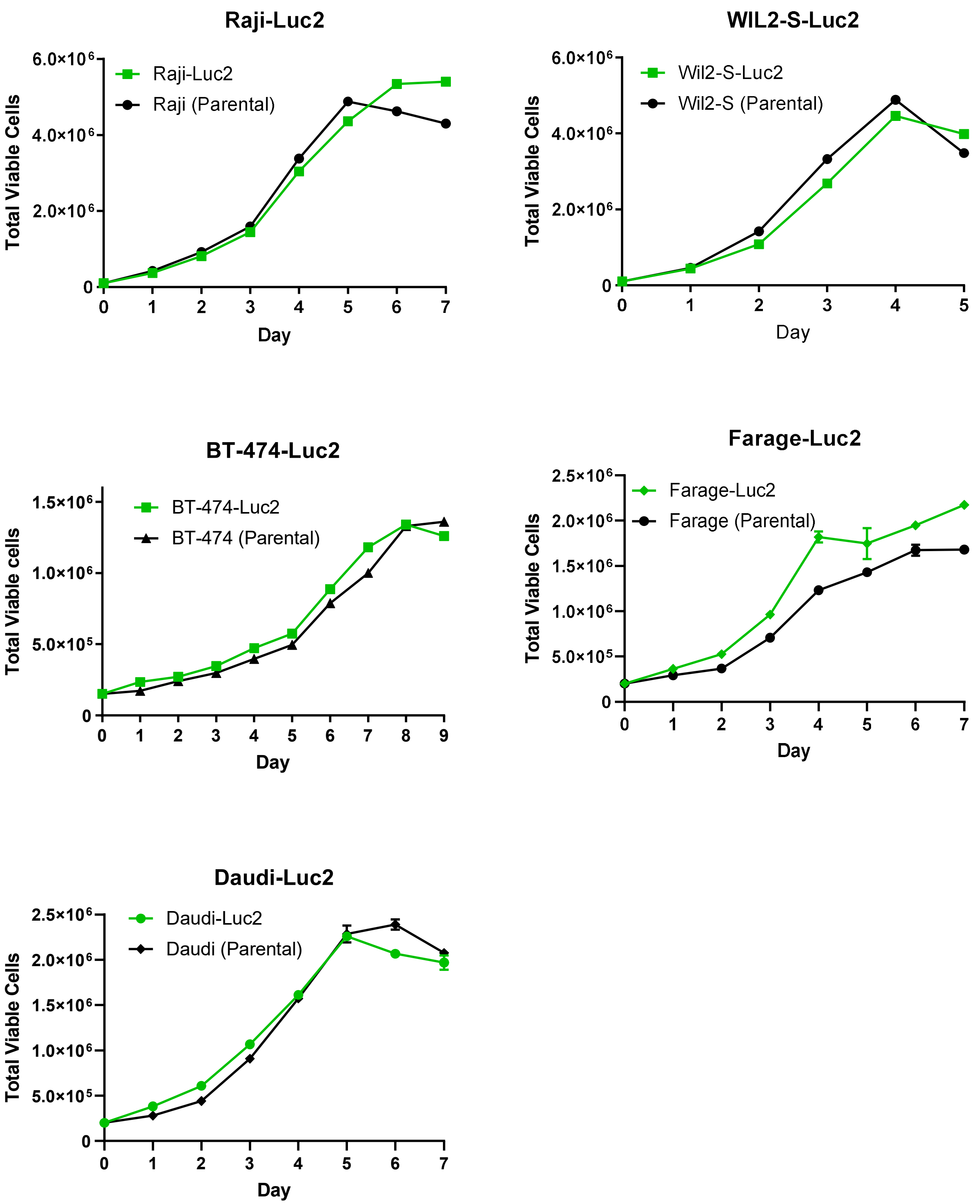

Figure S2: Characterization of CAR-T target cell lines: Growth Curve. 2 x 10^5 cells/mL were seeded into T25 flasks and cell counts were performed every day for 5, 7 or 9 days. The parental cell lines are shown in black, and the CAR-T target luciferase reporter cell lines are shown in green.

Supplemental Fig. S3

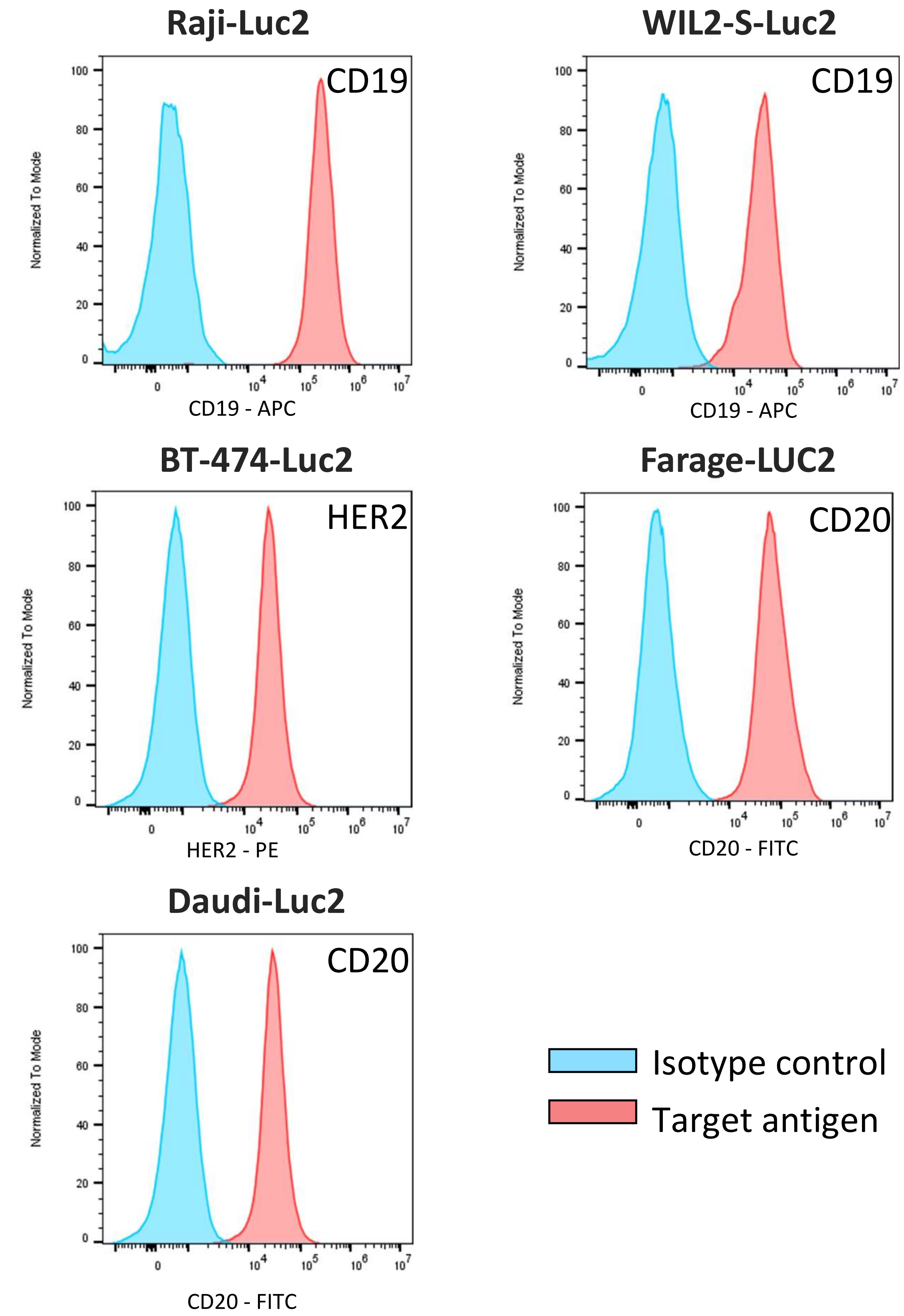

Figure S3 Characterization of CAR-T target cell lines: Antigen FACS analysis. Flow cytometry was performed to assess the CAR-T target antigen expression levels of CD19, CD20, and HER2 (pink) on the tumor cell lines compared to isotype controls (blue).

Supplemental Fig. S4

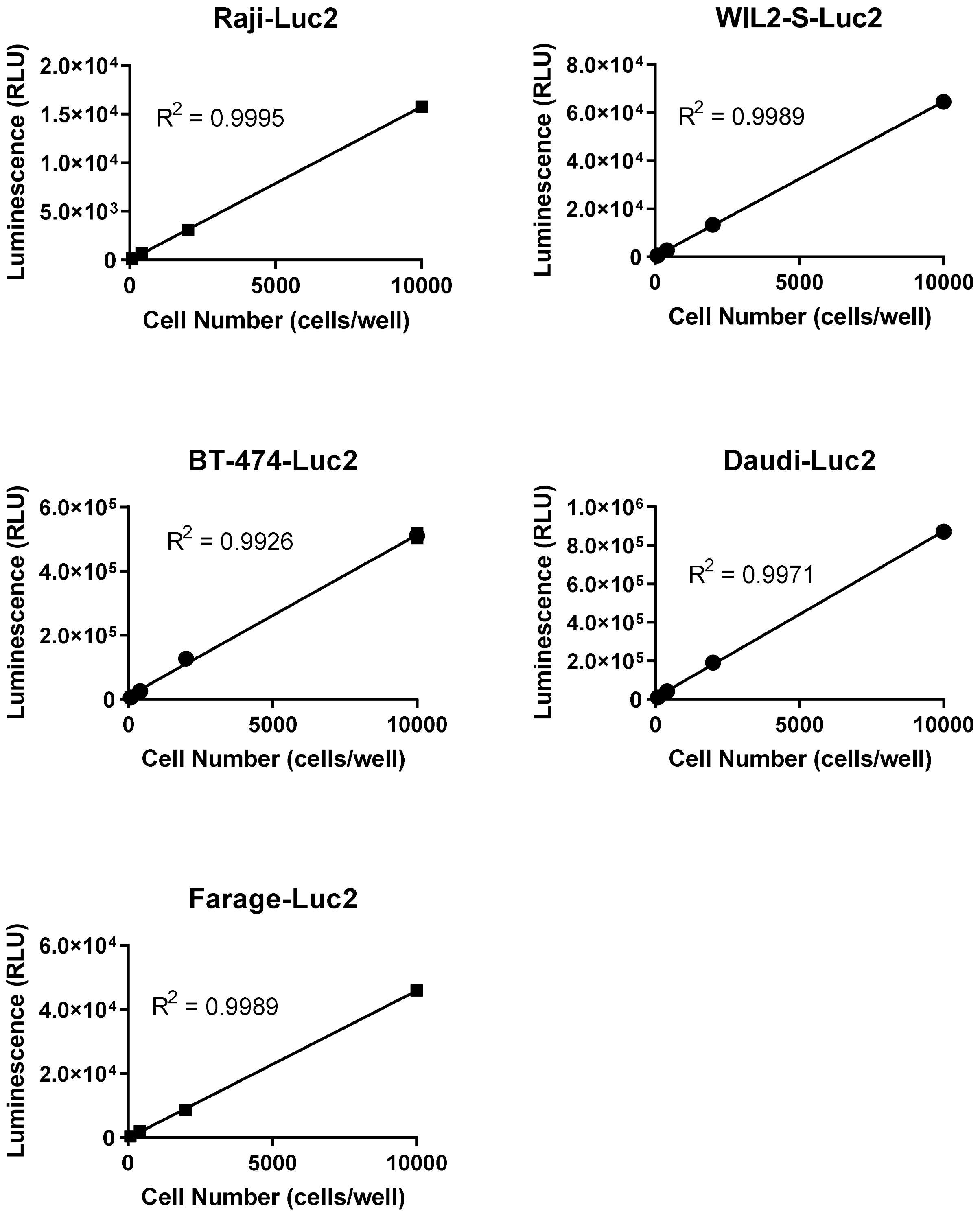

Figure S4: Characterization of CAR-T target cell lines: Luciferase assay. Luciferase expression was measured using Bright-Glo™ reagent and a luminescence plate reader for increasing numbers of cells. The results show a linear correlation between bioluminescence intensity and cell number.

Supplemental Fig. S5

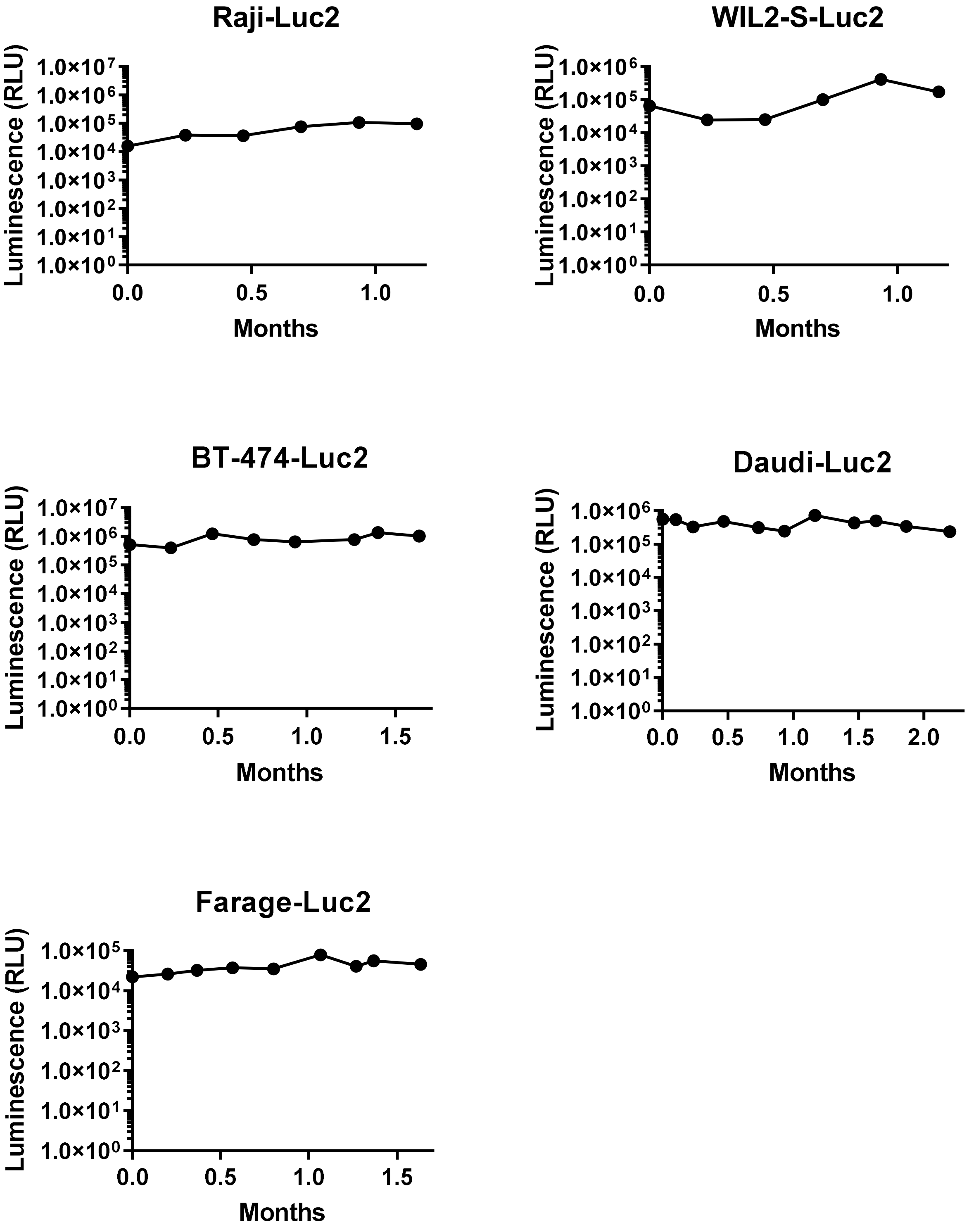

Figure S5: Characterization of CAR-T target cell lines: Luciferase activity stability. To verify the stability of luciferase expression, the cells were maintained in culture for 30 population doublings. Luciferase expression was monitored every week.
